# Case of Prolapse of Funis and Craniotomy

**Published:** 1861-02

**Authors:** F. N. Smith

**Affiliations:** La Harpe, Illinois


### CASE OF PROLAPSE OF FUNIS AND CRANIOTOMY.

By F. N. SMITH, M. D., of La Harpe, Illinois

I was called to see Mrs. W——, twenty-two years of age, in her second confinement, about noon of Friday, the 9th of November last. She was having pains at regular intervals, and tolerably severe. On examination, I found the head presenting; but through the membranes, yet unbroken, I could feel what I feared was the funis, ready to descend as soon as they should rupture. The first stage was about completed. A few pains more sufficed to burst the membranes, and what I feared, was now a dread reality. The cord came down nearly to the external parts. I immediately set about trying to place it above the head, in such a manner that it should be retained there. The method proposed by Dr. Meigs—a loop made of a short bit of tape placed around the cord, and a silver male catheter with which to place it up—

was ineffectual. I could not approximate it by means of my hand. An old midwife who was present, learning my perplexity, insisted that as her hand was smaller than mine, she could succeed perhaps. I permitted her to try. She succeeded, however, no better than I did. The child's head now completely filled the upper strait; still there was quite a strong pulsation in the cord. From indications, I began to fear, in addition to prolapsed funis, that there would be difficulty in accomplishing delivery, from the size of the head being so much greater than the capacity of the pelvis. I therefore sent to Blondinsville, a distance of three and a half miles, for Dr. Huston to assist me in the case, and to La Harpe, five miles for obstetric instruments. Dr. H. arrived about nine o'clock in the evening. For several hours—at least four—previous to this, labor had been making no progress, though the pains had been very energetic all the time. The doctor made an examination, and we decided that further effort at reduction of the cord would be useless. He thought we had better leave the labor to nature a while longer. This was agreed to; we waited till midnight, making occasional examinations, with reference to ascertaining what progress labor was making, if any. Nothing had been gained, and the agonizing pains were begining to exhaust the strength of our patient. Pulsation in the cord had ceased. Believing that further delay was not only useless, but jeopardizing the life of the patient, we now made preparations for the operation of craniotomy. The woman was placed partly on her left side across the bed, her hips resting on its edge, and her feet supported by chairs. I used Smellie's Scissors, which being warmed and oiled, I guarded with my index and middle finger, and carried its point to the head of the child, a little to one side of the suture. I directed my assistant to press the instrument, till I found it had entered up to the shoulder-stops, so called. The handles were then drawn asunder sufficiently far, as I thought, to make an incission, that would admit the crotchet. The scissors were withdrawn, and the crotchet introduced, carefully guarding as before. As soon as a pain came on, I made moderate traction, guarding the portion of skull hooked into with the instrument.

Finding that moderate traction did no good, I made it firmer and firmer, till the portion of cranium upon which I was drawing gave way. The position of the crotchet was changed, and I again got it engaged. After firm and steady traction had been made for some time, the cranium once more gave away. I then gave the instruments into the hands of Dr. Huston. He succeeded about as I had done. Although both of us had used a great deal of force, in assisting the efforts of the patient, very little had been gained. We had now the skull so broken up, that we could no longer use the crotchet. After a little rest and consultation, in which Dr. H. gave it as his opinion, that there was some deformity, which prevented the delivery of the child, and which he feared from indications could not be overcome, I proceeded with my hand, used as a blunt hook, to make all the traction in my power, during the presence of pains. The walls of the cranium, of course, had become to a considerable extent, collapsed. By means of my hand, I accomplished more in a short time, than we had effected with all our other efforts. I began to be encouraged. When my hand became tired and cramped, Dr. H. took my place. We attended in our efforts of this kind for perhaps an hour and a half, making slow but steady progress; and now, what was left of the head was in the world. By grasping the head, we could of course, aid the expulsive efforts of the woman to any extent we chose. But the strength of either of us individually could effect nothing at this juncture. We then attempted to bring down an arm; which was accomplished after a trial of some time. The effort requisite to bring down the other arm was not so great. The body was delivered with little delay. The hips became engaged, and were slowly and with a good deal of effort extracted. The placenta was speedily delivered, and there was but little hemorrhage. The child weighed nearly twelve pounds. Could it have been born alive, I judge it would have weighed at least thirteen pounds, perhaps more. It was about four o'clock Saturday morning, when the delivery was completed. The woman was not so much exhausted as I feared she would be. She inhaled considerable chloroform and ether, both before and after the operation; deeming it best to omit it at the time, that she might be perfectly conscious of any entanglement, or implication of the soft parts, and thus by her warning us, we could avoid anything serious in this way. She thought the chloroform saved her life, by enabling her to endure her pains; and I would say, that I believe the anæsthetic did not abate an atom of the energy of the pains, but rather added to their efficiency. She recovered without an unfavorable symptom. Her size is considerably under medium; and her pelvis not nearly so capacious as we should expect even in a woman of her weight. In her first confinement, the labor I was informed, was very protracted and severe, though her child was quite small, a girl, weighing not to exceed six pounds. It was born alive and healthy.

